# High-sensitivity cardiac SPECT system design with collimator-less interspaced mosaic-patterned scintillators

**DOI:** 10.3389/fmed.2023.1145351

**Published:** 2023-06-28

**Authors:** Rui Wang, Debin Zhang, Yifan Hu, Zhenlei Lyu, Tianyu Ma

**Affiliations:** ^1^Department of Engineering Physics, Tsinghua University, Beijing, China; ^2^Key Laboratory of Particle and Radiation Imaging, Ministry of Education (Tsinghua University), Beijing, China; ^3^Institute for Precision Medicine, Tsinghua University, Beijing, China

**Keywords:** myocardial perfusion imaging (MPI), single-photon emission computed tomography (SPECT), collimator, system design, mosaic pattern

## Abstract

**Purpose:**

Single-photon emission computed tomography (SPECT) is an important tool for myocardial perfusion imaging (MPI). Mechanical collimators cause the resolution-sensitivity trade-off in the existing cardiac SPECT systems, which hinders fast cardiac scan capability. In this work, we propose a novel collimator-less cardiac SPECT system with interspaced mosaic-patterned scintillators, aiming to significantly improve sensitivity and reduce scan time without trading-off image resolution.

**Methods:**

We propose to assemble a collimator-less cardiac SPECT with 7 mosaic-patterned detector modules forming a half-ring geometry. The detector module consists of 10 blocks, each of which is assembled with 768 sparsely distributed scintillators with a size of 1.68 mm × 1.68 mm × 20 mm, forming a mosaic pattern in the trans-axial direction. Each scintillator bar contains 5 GAGG(Ce) scintillators and 5 optical-guide elements, forming a mosaic pattern in the axial direction. In the Monte Carlo simulations, the in-plane resolution and axial resolution are evaluated using a hot-rod phantom and 5 disk phantoms, respectively. We simulate a cardiac phantom that is placed in a water-filled cylinder and evaluate the image performance with different data acquisition time. We perform image reconstruction with the expectation–maximization algorithm using system matrices derived from the simulation of a uniform cylindrical source filling the field-of-view (FOV). Besides, a 2-D prototype system is designed to demonstrate the feasibility of the collimator-less imaging concept.

**Results:**

In the simulation system, the sensitivity is 16.31% ± 8.85% in a 180 mm (Φ) × 100  mm (L) FOV. The 6-mm rods in the hot rod phantom and the 5-mm disks in the disk phantom are clearly separable. Satisfactory MPI image quality is achieved in the cardiac phantom study with an acquisition time of 30 s. In prototype experiments, the point sources with an 8 mm center-to-center distance are clearly separable at different positions across the FOV.

**Conclusion:**

The study reveals a promising approach to high-sensitivity SPECT imaging without a heavy-metal collimator. In cardiac imaging, this approach opens the way to a very fast cardiac scan with good resolution. Further works are ongoing to build a practical 3-D imaging system based on the existing design.

## 1. Introduction

Coronary artery disease (CAD) is the third leading cause of mortality worldwide associated with 17.8 million deaths annually (1). Myocardial perfusion imaging (MPI) is one of the most frequently used non-invasive diagnostic methods for assessing coronary blood flow. MPI identifies regional abnormalities in coronary artery blood flow and determines the physiological relevance to myocardial function and viability. Single-photon emission computerized tomography (SPECT) is the most commonly used imaging technique for MPI diagnosis (2). The typical imaging protocol involves intravenous injection of a radioactive blood flow marker (i.e., ^99m^Tc-sestamibi, ^201^Tl-chloride, or ^99m^Tc-tetrofosmin), tomographic data acquisition under stress and rest conditions, and volumetric/regional myocardial uptake analysis (3, 4).

Cardiac SPECT imaging performance has been rapidly improving over the last 10 years. With a conventional dual-head SPECT scanner, the detection efficiency is around ~130 cps/Mbq and the image resolution is ~15.3 mm (FWHM) without the resolution recovery techniques according to the National Electrical Manufactures Association NU-12001 protocol, where the standard orbit radius for dual-head SPECT is 15 cm (5, 6). The typical injected dose is 15–30 mCi for ^99m^Tc-sestamibi, a most widely used tracer in MPI imaging (7). The acquisition time is around 15 min with dual-head cameras for nearly all the imaging protocols. With the introduction of new detector technology, optimized collimator, and system design dedicated for imaging the heart region, improved performance has been successfully achieved in terms of reduced scan time, lower radiation dose, and higher imaging resolution. Representative systems include the NM 530c® SPECT(General Electric) and D-SPECT® (Spectrum Dynamics). Both systems utilize the new cadmium zinc telluride (CZT) detectors to achieve better energy resolution (~6%@140 keV) and intrinsic spatial resolution (2.5 mm) (8–11). A new diverging–converging (SMARTZOOM) collimator in IQ·SPECT® (Siemens) is developed that increases the photon sensitivity (6, 12, 13). Other dedicated system configurations optimized for cardiac imaging are also proposed, such as Cardius® 3XPO system (Digirad) and CardiArc® scanner. Cardius® system employs a cardio-centric orbit instead of a body-centered orbit with a compact triple-headed geometry moving around a sitting patient. In this case, the scanners are placed close enough to the chest for image quality enhancement (14). CardiArc® scanner applies a stationary curved scanner head and a unique curved lead sheet with a series of slits rotating back and forth during data acquisition (14, 15). Other dedicated cardiac SPECT systems developed in research labs are also reported (16–23), and some are commercially available (24, 25). Besides, the coded-aperture collimator has been developed for small animal imaging, which can be regarded as a highly multiplexed pinhole collimator achieving higher sensitivity while maintaining good image resolution (26). However, the performance of coded-aperture SPECT is still limited by the resolution-sensitivity trade-off. In Table 1, we summarize the performance of representative dedicated cardiac SPECT systems in comparison with conventional general-purpose systems. In general, dedicated cardiac SPECT systems achieve ~0.03–0.1% of sensitivity and ~ 6–10 mm of image resolution with an acquisition time of 4.5 to 10 min (5, 6, 15, 27, 28).

**Table 1:** System performance of the dedicated cardiac SPECT systems as compared with the conventional dual-head system.

Name	Conventional	D-SPECT	NM 530c	IQ-SPECT	Cardius
Detector	NaI/PMT	CZT	CZT	Dual NaI/PMT	pixelated CsI(Tl) with photodiodes
Collimator	Parallel-hole	Parallel-hole	Multi-pinhole	SMARTZOOM	Cardiac fan-beam
Acquisition time (min)	16	6	10	4.5	7.5
Sensitivity (Cps/MBq)	130	850	460	390	324
Resolution (mm)	15.3	8.6	6.7	15	9.2

Fast scanning is critical for cardiac imaging. However, existing cardiac SPECT scanners require a certain mechanical collimator to form projection, which causes substantial photon loss and tempers sensitivity. Compared to the conventional parallel-hole collimator, although focusing collimators such as multi-pinhole, slit-slat, or cardiac fan-beam collimators effectively improves the resolution-sensitivity trade-off in the heart FOV, the inevitably dramatic photon loss on the mechanical collimators still lead to an overall low sensitivity for the imaging system and strongly limit the fast imaging capability.

Compton camera is one collimator-less imaging method that can improve system sensitivity significantly. Compton-camera has been used in small animal imaging studies with millimeter-level resolution (29–31). However, it has not been applied for cardiac imaging in the human body due to the limited position resolution for far-field imaging. Also, the Compton camera is not suitable for low-energy (<200 keV) gamma source imaging (32).

Recently, our lab has proposed a novel collimator-less gamma camera design using a metal-free 3-D position-sensitive scintillator detector. With the interspaced mosaic-patterned scintillators, one scintillator is naturally collimated by other scintillators, and the detector response is sensitive to the directional position of the incoming gamma ray, providing sufficient information for radiation source imaging in a 4π field-of-view (FOV). We have achieved fast directional gamma-ray imaging with a high-sensitivity portable collimator-less gamma-camera design for nuclear security applications (33). Besides, we have also developed a new mosaic-patterned gamma camera embedding scintillators and heavy metal elements, achieving better imaging performance for middle- and high-energy sources (34).

In this work, we aim to improve the sensitivity of cardiac SPECT imaging to a new level by extending the collimator-less gamma imaging technique from 2-D directional imaging to 3-D tomographic SPECT imaging. We propose to assemble a ring-shaped system with multiple mosaic patterned, uncollimated scintillator blocks that allow a flexible system design. We perform Monte Carlo simulations to evaluate the resolution and sensitivity performance and investigate the cardiac imaging feasibility with an anthropomorphic phantom. We test the imaging feasibility with a 2-D experimental prototype.

## 2. Materials and methods

### 2.1. Collimator-less imaging concept and simulated cardiac SPECT system

#### 2.1.1. Collimator-less imaging concept

**Figure 1:**
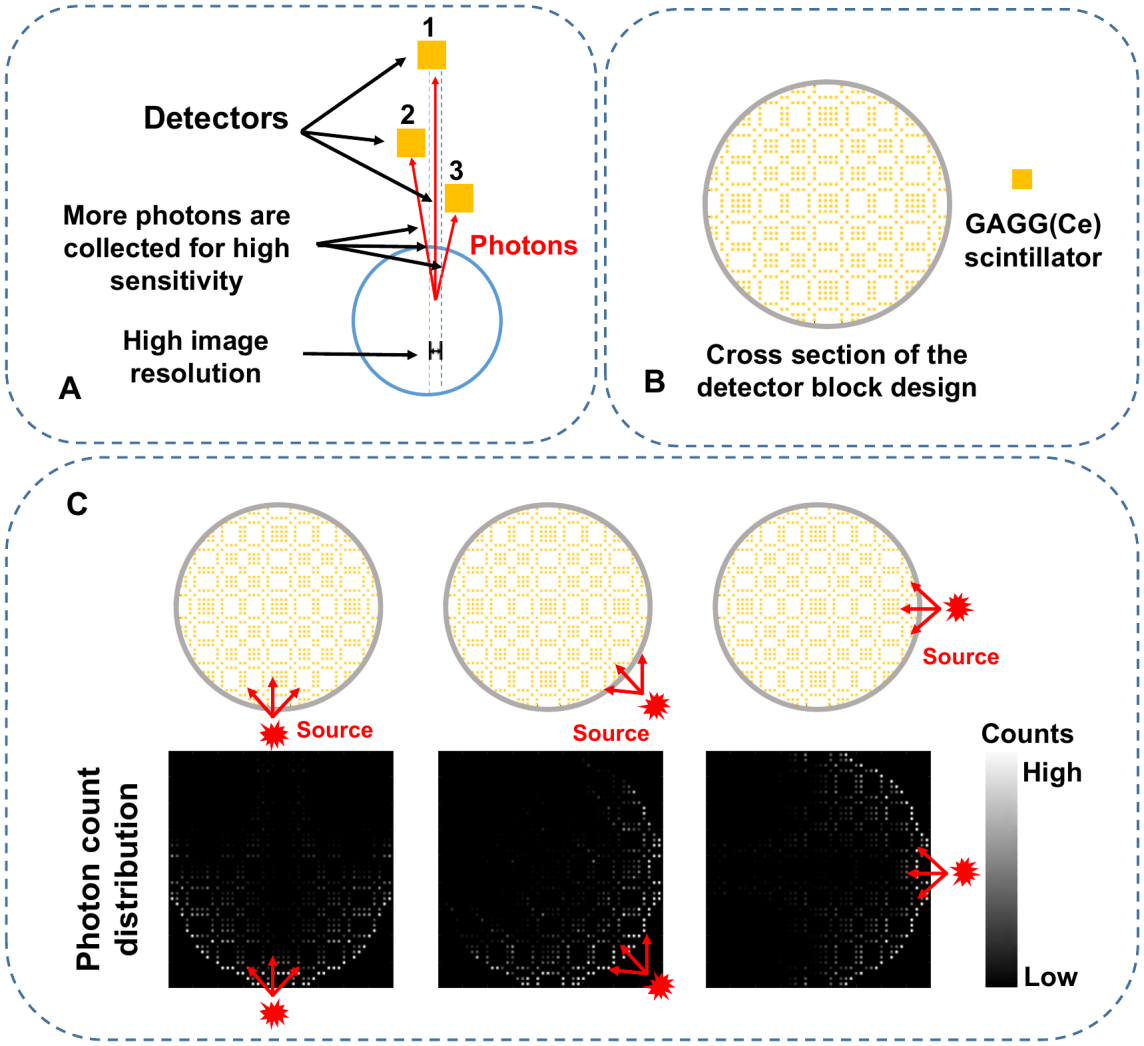
**(A)** Schematic diagram of the proposed self-collimation concept with three individual detectors. The red lines indicate gamma photons detected by the specific detector in the blue dashed region. **(B)** 2-D mosaic scintillation design in the trans-axial plane. **(C)** Schematic diagram of the photon counts distribution. The red-arrow lines represent the paths of the incoming photons from three different directions.

The collimator-less imaging concept is shown in Figure 1A. Detectors 2 and 3 are separately placed between the source and detector 1. In this way, the photon absorption on detectors 2 and 3 naturally forms photon collimation for detector 1. Besides, the absorbed photons on detectors 2 and 3 are also contributable to the image formation process, leading to significant sensitivity improvement compared to conventional mechanical collimation.

Figure 1B shows the cross-section of a complete collimator-less detector block. We propose to place multiple individual GAGG(Ce) scintillators inside the block to form a mosaic pattern in the trans-axial plane. Compared to our previously used mosaic-patterned detector for a planar gamma imager (33), in this work, we use a sparser mosaic pattern to spread the incoming gamma photons over more detector elements. We also choose a circular-shaped detector block instead of a square-shaped block in the SPECT imaging system to reduce gaps between detectors. As shown in Figure 1C, when a gamma source is placed at different positions, the accumulated photon count distribution (i.e., the projection) is dependent on the source position.

#### 2.1.2. System design

**Figure 2:**
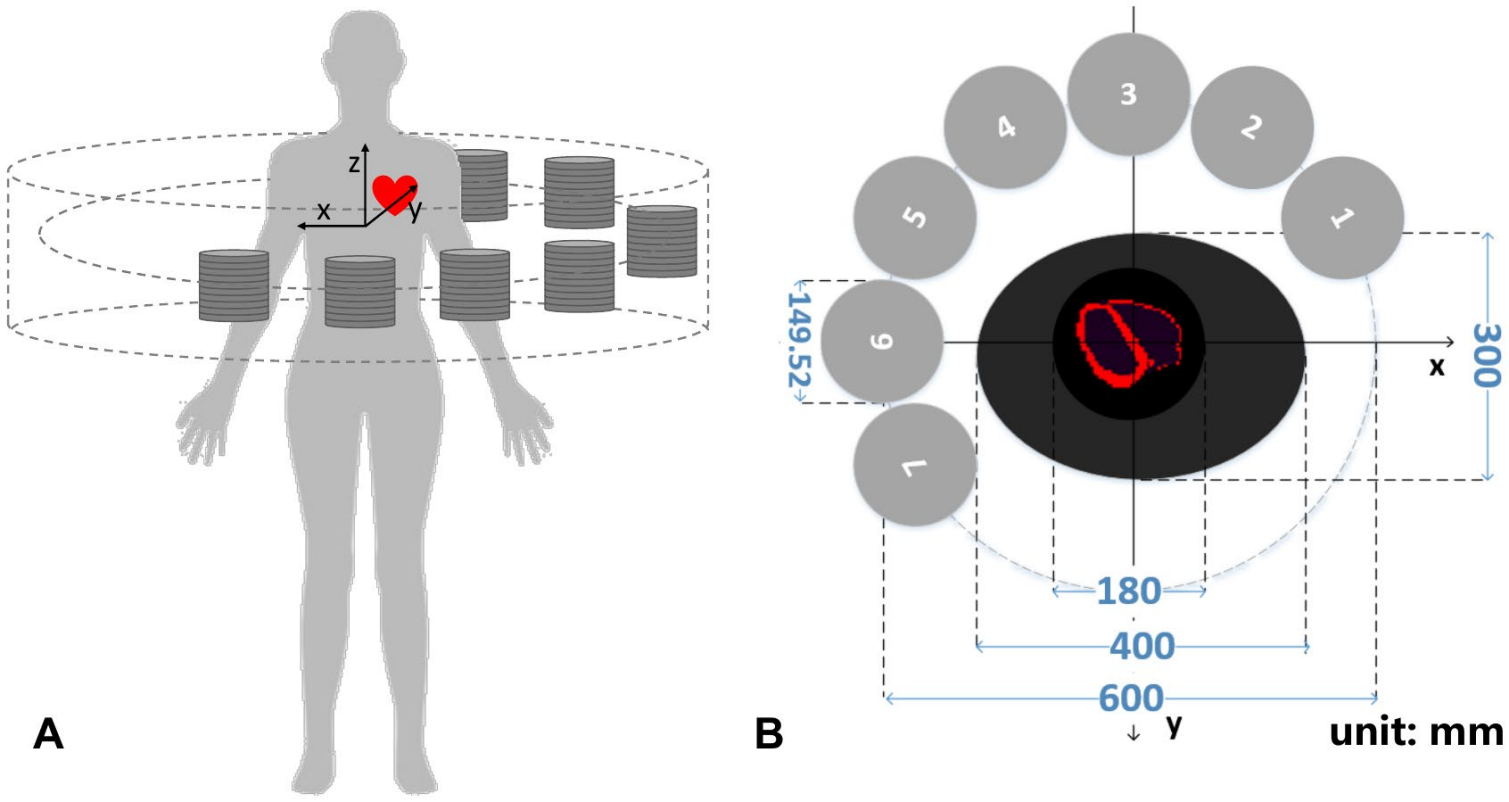
**(A)** 3-D schematic diagram and **(B)** transverse cross-section of the cardiac imager system (unit: mm).

Figure 2A illustrates a designed collimator-less cardiac SPECT system. We place 7 detector modules surrounding the patient’s body to form a half-ring geometry. As shown in Figure 2B, the intended FOV to cover the heart region is 180 mm (Φ) × 100 mm (L), which is suitable for the majority of patients under a survey of cardiac and thoracic anatomy of cardiac patients (20). The system is designed to accommodate the body contour, which is mimicked by an elliptical region with the size of 400 mm (long axis) × 300 mm(short axis) in the cross-sectional plane. The 7 detector modules are evenly spaced from the 60° right anterior angular position to the 30° left posterior position. Their geometrical centers are on a 600-mm-diameter circle.

#### 2.1.3. Detector module design

**Figure 3:**
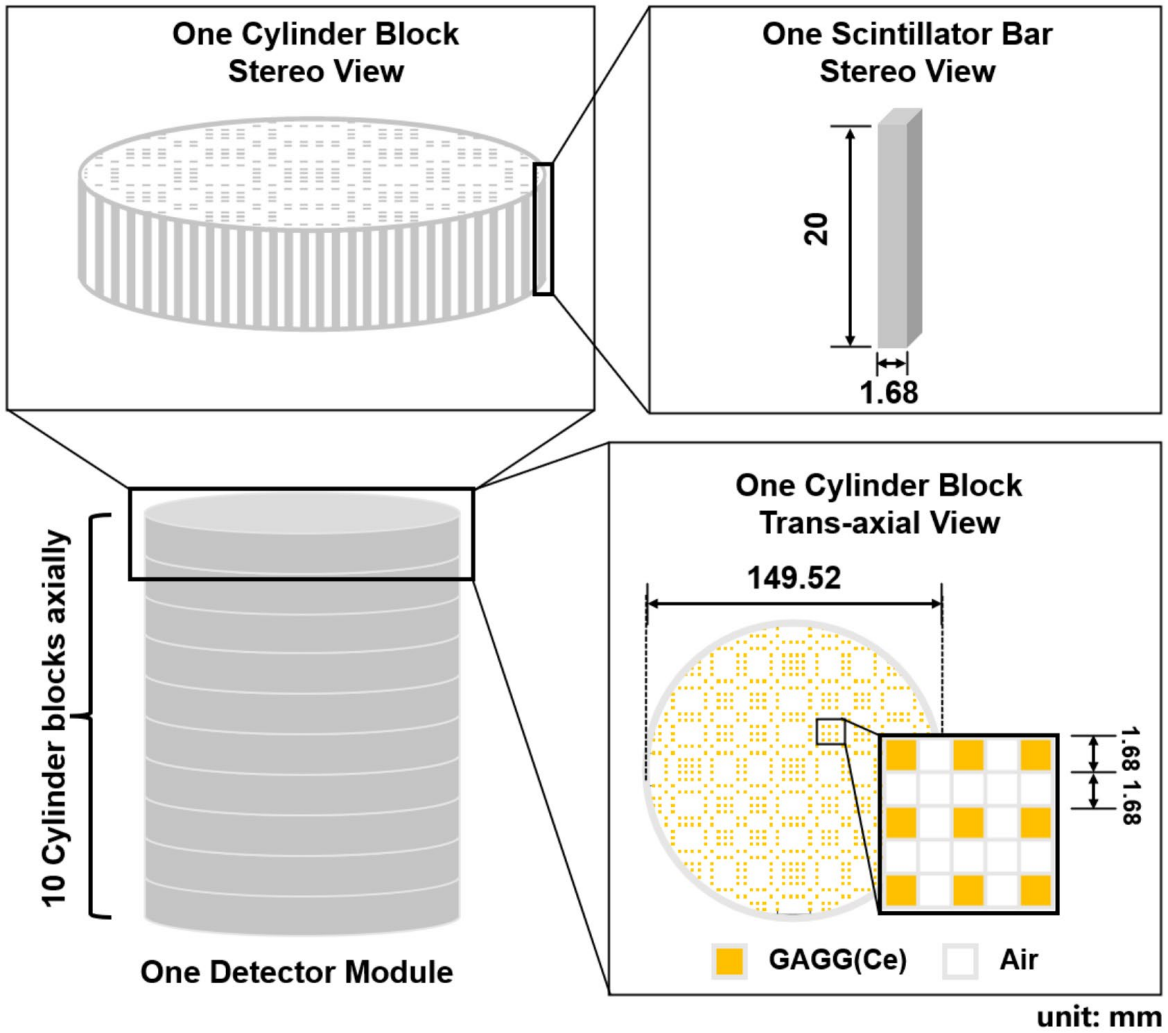
Stereo view and trans-axial view of one detector module (unit: mm).

Figure 3 shows the geometrical setup of a detector module. Each module contains 10 detector blocks that are stacked axially. The size of each detector block is 149.52 mm (Φ) × 20 mm (L). Each cylindrical detector block consists of 768 GAGG(Ce) scintillator bars with a size of 1.68 mm × 1.68 mm × 20 mm. In each block, the scintillator bars form a mosaic pattern as shown in the trans-axial view of Figure 3.

**Figure 4:**
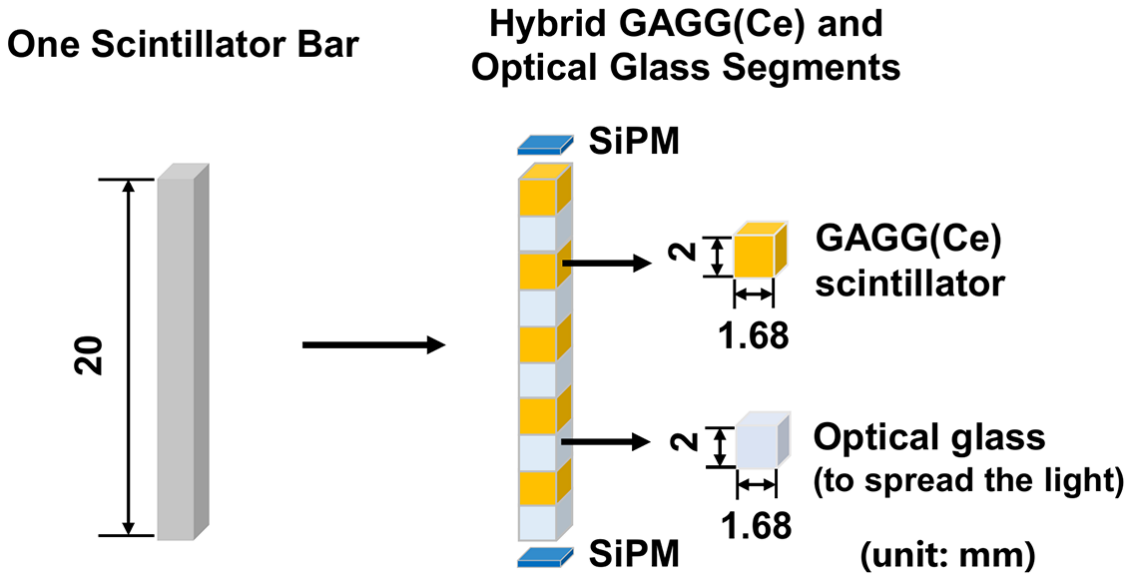
Schematic diagram of hybrid GAGG(Ce) and optical glass segments forming the mosaic pattern in the axial direction (unit: mm).

We propose to assemble a mosaic-patterned scintillator bar by interlacing five 1.68 mm × 1.68 mm × 2 mm GAGG(Ce) scintillator and five optical-guide segments (k-9 glass, Epic Crystal, China) as shown in Figure 4, where each scintillator is glued to the adjacent k-9 glass segment. The linear attenuation coefficient of the k-9 glass for 140 keV photons is 0.399 cm^−1^, which is significantly lower than that of the GAGG(Ce) scintillator (4.746 cm^−1^). The refraction indexes for the k-9 glass and the GAGG(Ce) scintillator are 1.5 and 1.9 respectively, which are close to each other. Such a design both allows an interspaced, mosaic-patterned spatial structure of the GAGG(Ce) scintillators and enables efficient optical-photon transportation for each scintillator to the two silicon photo-multipliers (SiPM). The entire detector block contains 38,400 spatially separated scintillator segments. In the cross-sectional plane, each scintillator bar is wrapped with total-reflective materials. The space between each scintillator bar is simply filled with air. Therefore, the electric signal readout is independently operated for each scintillator bar with two SiPMs on both ends.

#### 2.1.4. Monte Carlo simulations

We evaluate the system performance through Monte-Carlo simulations with the GATE v8.0 package (35). We model the half-ring cardiac imaging system in Sections 2.1.2 and 2.1.3 and simulate the transportation and detection process for 140 keV gamma photons to mimic ^99m^Tc labeled tracers. We set an intrinsic energy resolution of 20% at 140 keV for the GAGG(Ce) scintillators based on our previous experimental measurement (33) and acquire the data in a 112–168 keV energy window. In the simulations, the position of each event is determined by the scintillator element within which the maximum deposited energy is recorded.

We evaluate the imaging performance using three types of phantoms, including a hot-rod phantom to test the in-plane resolution performance, 5 disk phantoms to test the axial resolution performance, and a 3-D cardiac phantom to mimic the MPI imaging scenario. In cardiac imaging simulations, we place a water-filled cylindrical attenuator (Φ = 200 mm, H = 100 mm) in the FOV to account for the photon scattering and attenuation effects during projection data acquisition. No water-filled attenuator is applied in the hot-rod and disk phantom studies. We perform multiple rounds of simulations for both hot-rod and cardiac phantoms with different activity and acquisition time combinations during the image formation process.

The projection data are defined by concatenating the record counts in the detector elements of all the seven cylinder-shaped scintillator blocks into a single vector.


(1) $$Y_{simu}={\left[y_{1,1},y_{1,2},\ldots ,y_{1,n},y_{2,1},y_{2,2},\ldots ,y_{2,n},\ldots ,y_{m,1},y_{m,2}\ldots ,y_{m,n}\right]}^{T},$$

Where $y_{k,l}$ is the number of events acquired by detector element l on detector module k . n = 768 (in trans-axial plane) × 50 (in axial direction) denotes the total number of scintillator bars in one detector module. m =7 denotes the total number of detector modules.

We perform long-time Monte Carlo simulations to derive system matrices for the three phantom studies (36). A uniform ^99m^Tc cylindrical source (180 mm (Φ) × 100 mm (L)) filling the FOV is simulated for all three phantom studies. In the cardiac phantom study, we place the water-filled cylindrical attenuator (Φ = 200 mm, H = 100 mm) in the FOV to account for the attenuation and scattering effect. In each study, we acquire around 2 × 10^12^ events so that the impact of noise is minimal. Each recorded list-mode event contains the emission and detection position information during the simulation process. The system matrix [ $A_{ij}$ ] representing the probability that a photon emitted from *j*th voxel detected in the *i*th detector bin is approximated by the total number of recorded events in the *i*th detector bin and emitted from *j*th voxel. Table 2 summarizes the geometry parameters, and the counting statistics of the simulated system matrices for three phantom studies.

**Table 2:** Summary of geometry parameters, counting statistics involved in generating the system matrices.

System configuration	FOV dimension	Voxel size	Number of voxels	Number of detector bins	Average recorded events per voxel
hot-rod phantom w/o water-filled attenuator	180 mm (Φ) × 100 mm(H)	2 mm × 2 mm × 2 mm	90 × 90 × 50	268,800	4.01 × 10^6^
disk phantom w/o water-filled attenuator	180 mm (Φ) × 100 mm(H)	2 mm × 2 mm × 2 mm	90 × 90 × 50	268,800	4.01 × 10^6^
Cardiac phantom w water-filled attenuator	180 mm (Φ) × 100 mm(H)	3 mm × 3 mm × 3 mm	60 × 60 × 34	268,800	1.72 × 10^7^

A hot-rod phantom ( Φ = 180 mm, H = 100 mm) with a hot-rod size of 4, 5, 6, 7, 8, and 9 mm is utilized to evaluate the image resolution in the trans-axial plane. There is no background activity in the phantom, and the distances between the hot rods are twice their diameters. A total of 0.45 mCi activity is in the phantom, which is equivalent to 30 mCi tracer injection in the human body multiplied by 1.5% ^99m^Tc-sestamibi tracer uptake in the myocardium (37). Four imaging time cases are simulated, which are 20 min, 5 min, 1 min, and 20 s, and correspond to a total of 2.26 × 10^9^, 5.66 × 10^8^, 1.13 × 10^8^, 3.77 × 10^7^ events in the projection, respectively. Besides, considering the statistical noise in the Monte-Carlo simulation, we calculate the forward projection as a noise-free case to evaluate the optimal imaging performance. The forward projection is generated by direct multiplication of the system matrix and the 1-D scalar flattened from the 3-D volumetric hot-rod phantom image.

Five disk-shaped phantoms are applied to measure image resolution in the axial plane. Each phantom contains five cylindrical disks of equal size arranged axially at the same interval. The thickness of the cylindrical disk in each phantom is 4, 5, 6, 7, and 8 mm, respectively. The diameter of the cylindrical disk is 120 mm and the interval between two adjacent disks is twice the thickness of the disk for each phantom. We simulate a total of 0.45 mCi activity and 20 min acquisition time for each phantom.

In the 3-D cardiac phantom study, the tracer activity distribution in the myocardium is extracted from the XCAT phantom (38), where the relative uptake activity is 75 in the myocardium, 2 in the blood pool, and 2 in the coronary arteries and veins. Instead of a non-uniform attenuating medium in the XCAT phantom, the water-filled cylindrical attenuator (Φ = 200 mm, H = 100 mm) described before is applied around the cardiac region to simulate photon scattering and attenuation effects. Five imaging cases include the acquisition time of 20 min, 5 min, 1 min, 30 s, and 20 s each with 0.45 mCi tracer uptake in the heart, producing corresponding to a total of 2.26 × 10^9^, 5.66 × 10^8^, 1.13 × 10^8^, 5.66 × 10^7^, 3.77 × 10^7^ events in the projection, respectively. The voxel size of the reconstructed image is 3 × 3 × 3 mm^3^ for cardiac study, while the voxel size is 2 × 2 × 2 mm^3^ for hot-rod phantom and disk phantom study_._

#### 2.1.5. Reconstruction settings

We perform the image reconstruction with an ordered subset expectation maximization (OSEM) algorithm (39).


(2) $$f_{j}^{\left(k,q\right)}=\frac{f_{j}^{\left(k,q-1\right)}}{\sum_{i\in S_{q}}A_{ij}}\underset{i\in S_{q}}{\sum }A_{ij}\frac{y_{i}}{\sum_{l}A_{il}f_{l}^{\left(k,q-1\right)}},$$

where $f_{j}$ denotes the image value in $j^{th}$ pixel, $y_{i}$ denotes the number of photon counts detected in $i^{th}$ detector bin, *q* and *k* are indices of subsets and iterations, respectively. $S_{q}$ is the $q^{th}$ subset of the projection data. There are a total of 268,800 projection bins, which are grouped into 35 subsets. The algorithm uses parallel processing with the GPU card based on the MATLAB platform, running on a workstation with Intel Xeon Silver 4,110 CPU, 1 TB memory, and an NVIDIA TITAN Xp GPU card with 12 GB GPU memory. Each iteration takes 10 min, 10 min, and 5 min for hot-rod, disk, and cardiac phantom reconstruction.

In all cases, we empirically determine an iteration number that represents an optimal trade-off between resolution and noise. The iteration numbers for the hot-rod, disk, and cardiac phantom study are 50,10, and 50, respectively. We also apply a post-reconstruction Gaussian filter with empirically chosen parameters. Specifically, the Gaussian filter with a kernel size of 7 and FWHM of 2 mm is applied in hot-rod and disk image processing. The Gaussian filter with a kernel size of 7 and FWHM of 7 mm is applied in cardiac image processing.

### 2.2. Experimental prototype system

#### 2.2.1. Detector design

We perform proof-of-concept experiments with a mosaic-patterned detector block developed in our lab (33). The detector setup is different from the system design in Section 2.1. The aim of following experiments is to demonstrate the feasibility of performing collimator-less SPECT imaging, rather than validating the predicted performance in simulations.

**Figure 5:**
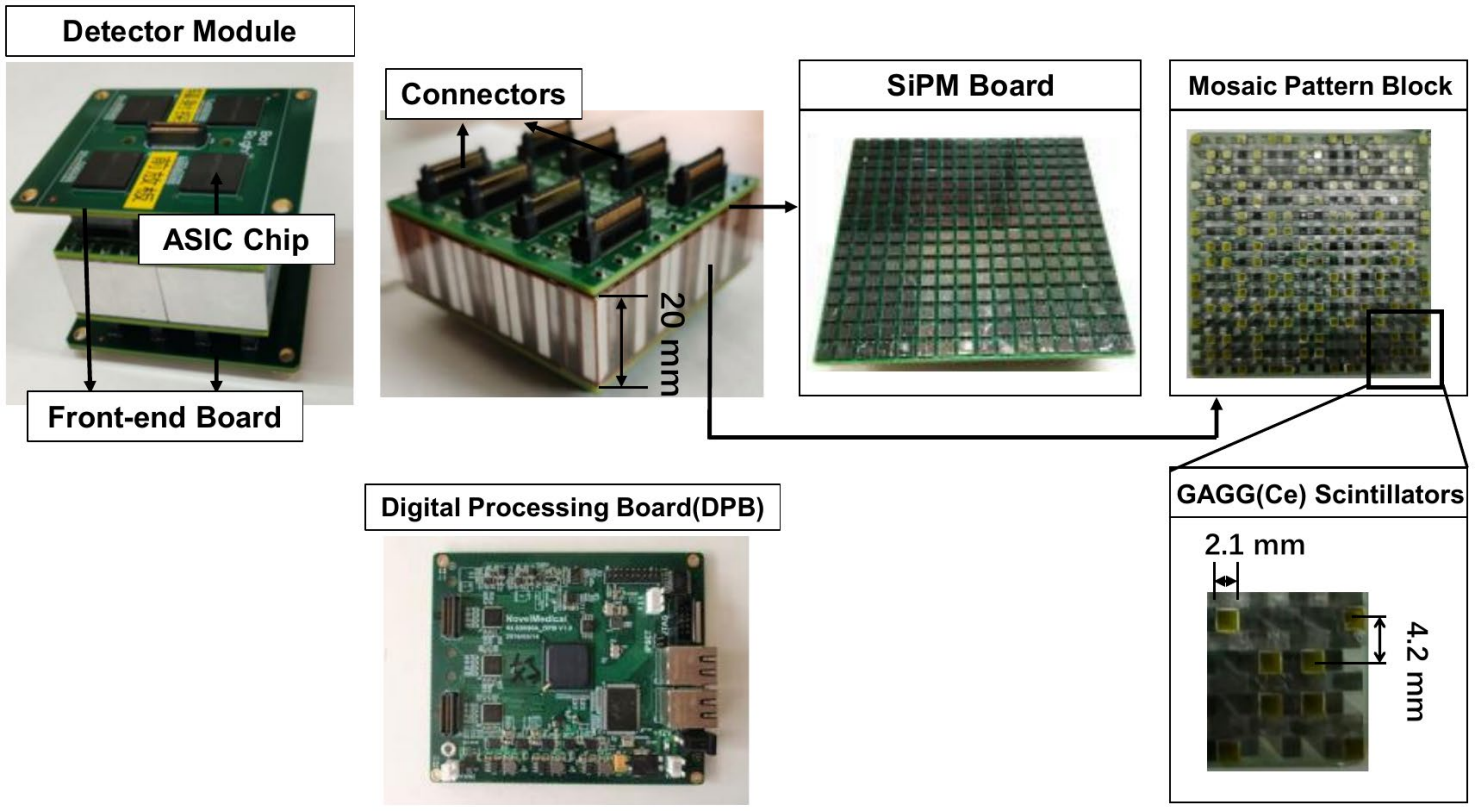
Experimental detector modules.

The detector block is shown in Figure 5. The size of the detector block is 67.5 mm × 67.5 mm × 20 mm. There are 128 spatially separated GAGG(Ce) scintillator (Epic Crystal, China) bars embedded in the plastic framework in one detector block. The size of each GAGG(Ce) scintillator bar is 2.1 mm × 2.1 mm × 20.0 mm, and the distance between two adjacent scintillator bars is 4.2 mm. Each scintillator is wrapped with a 0.15 mm thickness BaSO_4_ reflector (Epic Crystal, China). The mosaic-patterned scintillator block is optically coupled to two 16 × 16 SiPM boards (Onsemi, FJ30035) at both ends. Each SiPM board is connected to one front-end board with customized ASIC chips (40) that produce analog signals of 2-D position(X/Y) and energy(E) with in-chip anger-logic resistor networks. On the back of a single SiPM board, four self-developed ASIC chips are positioned, each generating one set of X, Y, and E signals for every recorded event. Subsequently, the X, Y, and E signals are digitized using A/D converters (AD9637, 12-bit, and 80 MHz sampling rates) located on the digital processing board(DPB) for data analysis.

#### 2.2.2. System configuration and gantry

**Figure 6:**
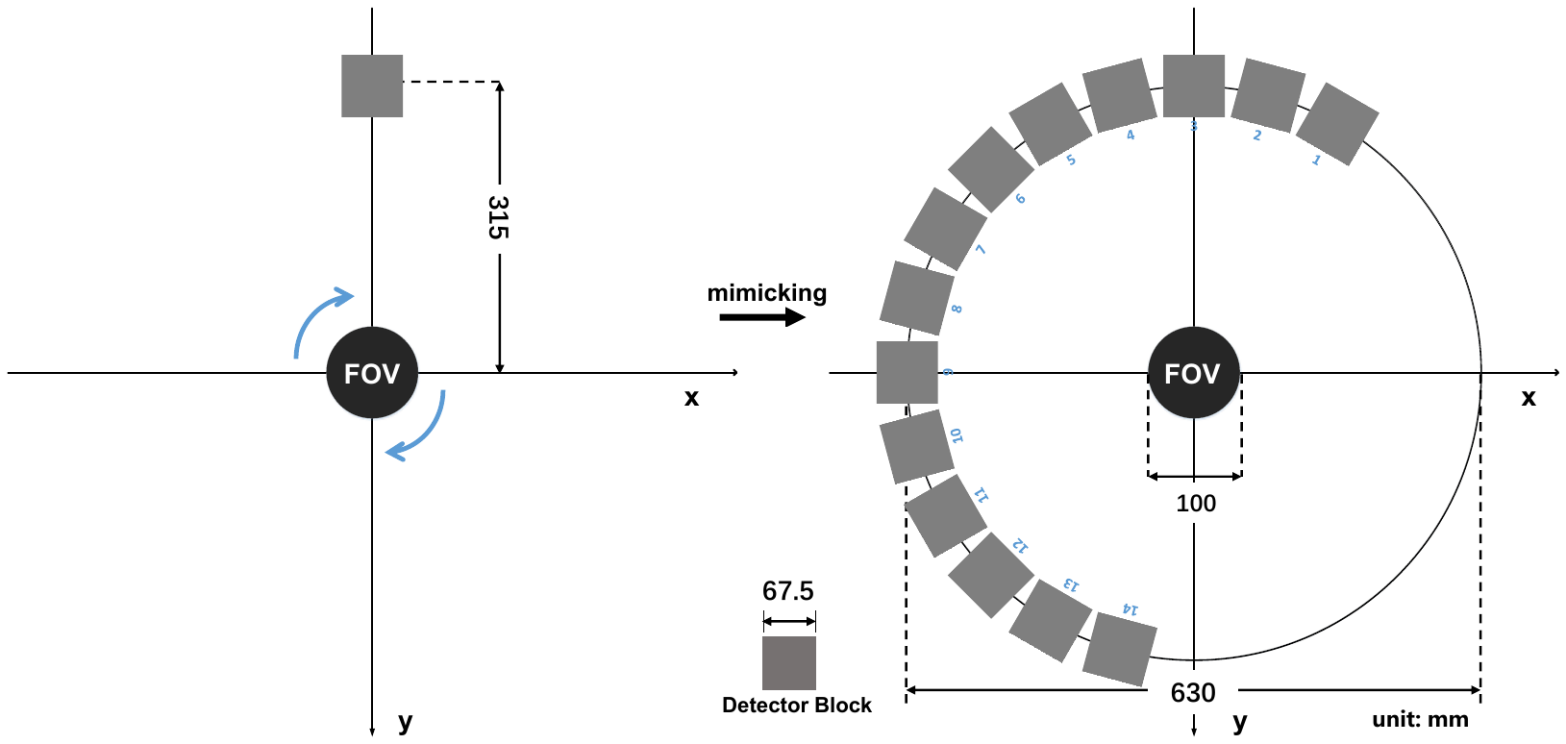
Transverse cross-section of the experimental prototype imager system (unit: mm).

As shown in Figure 6, We rotate a planar phantom about the central axis for 13 steps, which is equivalent to placing the detector at the 13 positions surrounding the object. In this way, we virtually define a half-ring imaging system with 13 detector blocks surrounding the 2-D FOV.

#### 2.2.3. Data acquisition and reconstruction settings

In the 2-D experimental case, the projection vector is also expressed as Eq. (1), where the number of detector modules is *m* = 13 and the total number of scintillators in one detector module is *n* = 128. The experimental system response matrix is $A_{\exp}$ ∈ ${\mathbb{R}}_{N\times M}$ , where *M* = 10^4^ is the total number of voxels in the FOV, and *N* = 128 × 13 is the total number of detector bins in the virtual system.

**Figure 7:**
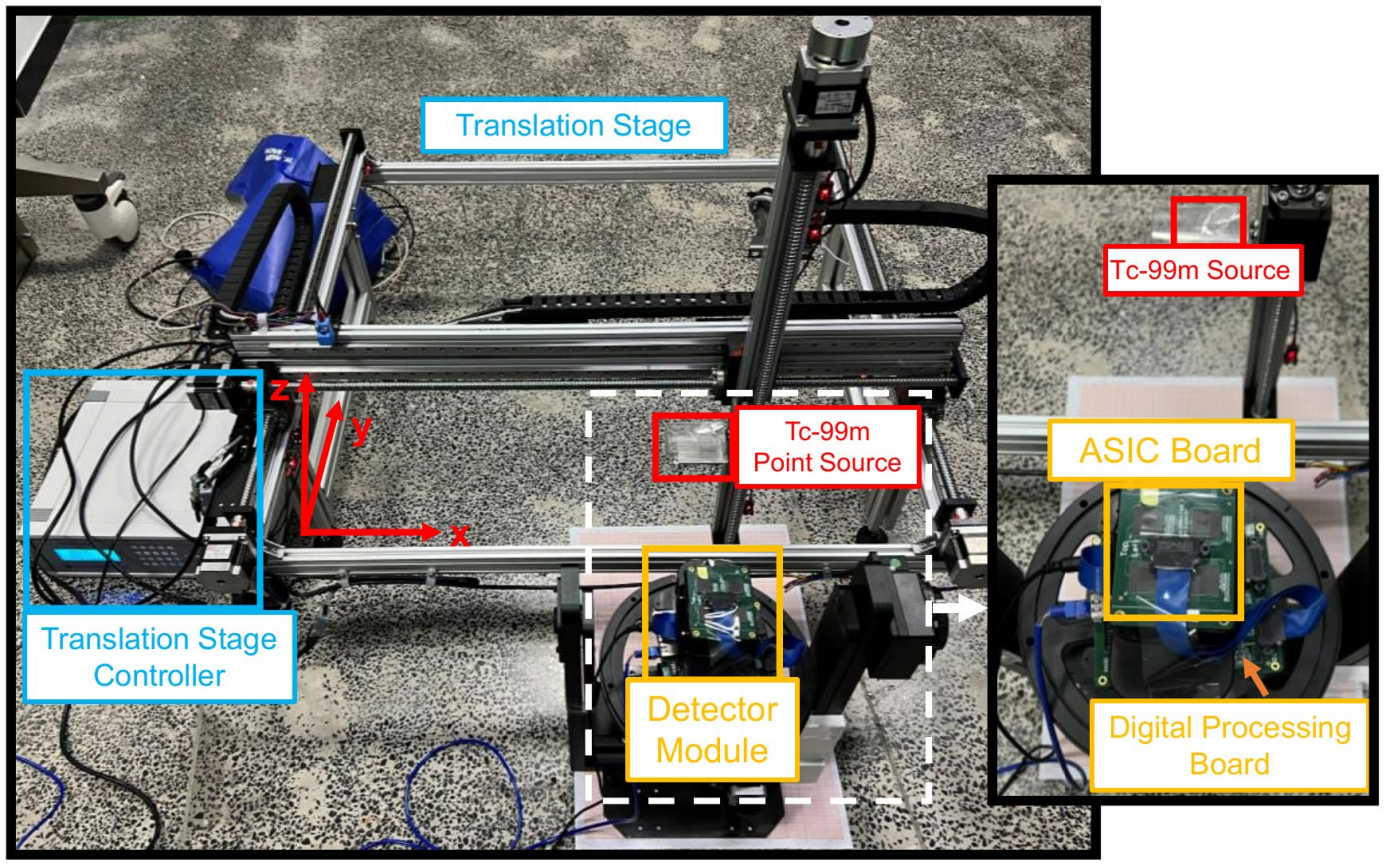
The experimental platform.

We use a ~ 9.7 mCi ^99m^Tc point source with a diameter of 0.69 mm to measure the system matrix of the single detector module. As shown in Figure 7, we place the point source on a holder that is fixed to a translation stage. We measure a single-detector system matrix with a FOV size of 100 mm × 100 mm and a voxel size setting of 3 mm × 3 mm. There are 35 × 35 measurement positions during the experiment. Around 1.4 M events are collected with 10s acquisition in each measurement position. The FOV of the single-detector system matrix is expanded from 35 × 35 grids (x = −51: 3: 51 mm, y = −51: 3: 51 mm) to 103 × 103 grids (x = −51: 1: 51 mm, y = −51: 1: 51 mm) by cubic spline interpolation. Then, we calculate the system matrix for the half-ring-system from the single-detector system matrix according to rotational geometry symmetry.

To evaluate the imaging performance of the experimental prototype, we conduct a point-source-based experiment. The diameter of the point source is 0.69 mm. To mimic the 13-detector system in Figure 6 based on one detector block, we calculate the relative location of the source and each detector block, and place the point source at these locations to acquire projection data, respectively. Then, all 13 projection data vectors are concatenated to form the projection of the half-ring system for one point source in the FOV.

To evaluate the system resolution when imaging multiple point sources at a certain distance, we place a point source at different positions and acquired a list-mode dataset at each position. We precisely know the distance between each position by moving the point source from one position to the other using the translational stage. We generate the projection data by binning the list-mode events at all the individual positions into a single projection. This generated projection is equivalent to the one measured with multiple point sources at different positions. We evaluate two cases, a two-point-source phantom with an 8-mm center-to-center distance and a 2-by-2 point source array with an 8-mm center-to-center distance. In the two-point source experiment, we evaluate three imaging cases with equivalent acquisition times of 240 s, 24 s, and 2.4 s using 0.45 mCi total activity of point sources. In the 2-by-2 point source experiment, the total acquisition time for all the point sources is around 30 min.

We apply the maximum likelihood expectation maximization (MLEM) algorithm, i.e., using one subset in Eq. (2) for image reconstruction in the experiments. Similar to the simulation studies, early-stop iterations and the post-reconstruction Gaussian filter are empirically chosen as well.

## 3. Results

### 3.1. Simulated cardiac SPECT system

**Figure 8:**
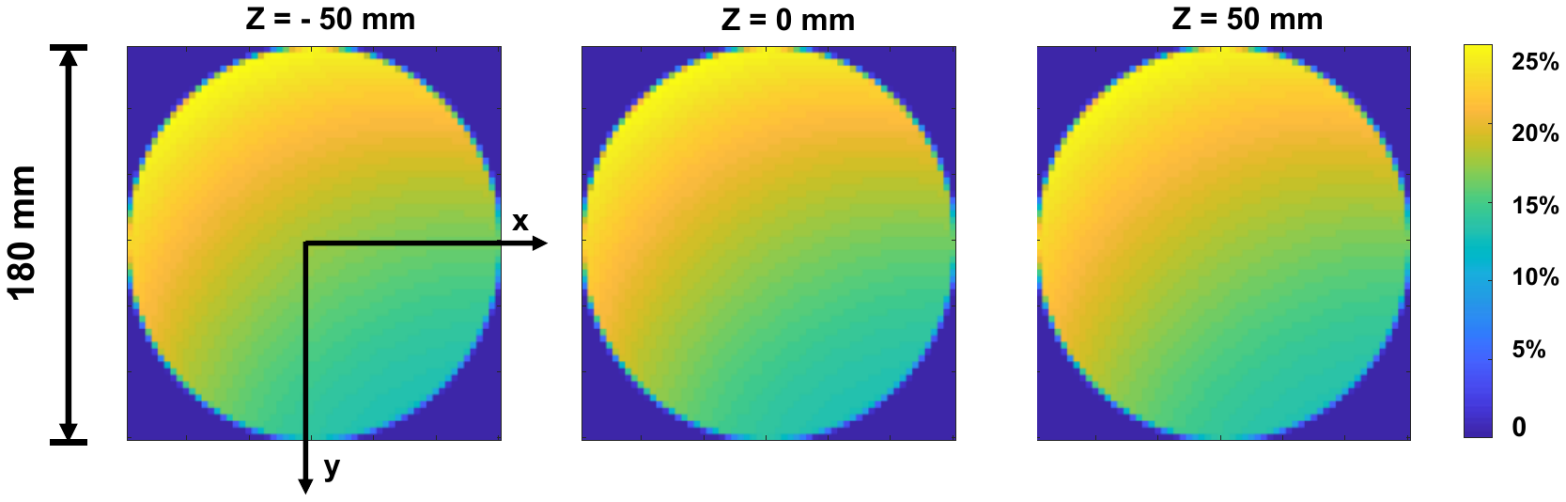
Sensitivity map of top-slice, medium-slice, and bottom-slice in the FOV (Φ = 180 mm, H = 100 mm).

The sensitivity was determined as the number of recorded events detected from the detector bins divided by the number of emitted photons in each voxel during the Monte-Carlo simulation. Figure 8 shows the sensitivity map of the simulated cardiac SPECT in three axial slices. The average measured sensitivity in the whole FOV is 16.31 ± 8.85%. There is no big difference between the sensitivity maps at different axial positions.

**Figure 9:**
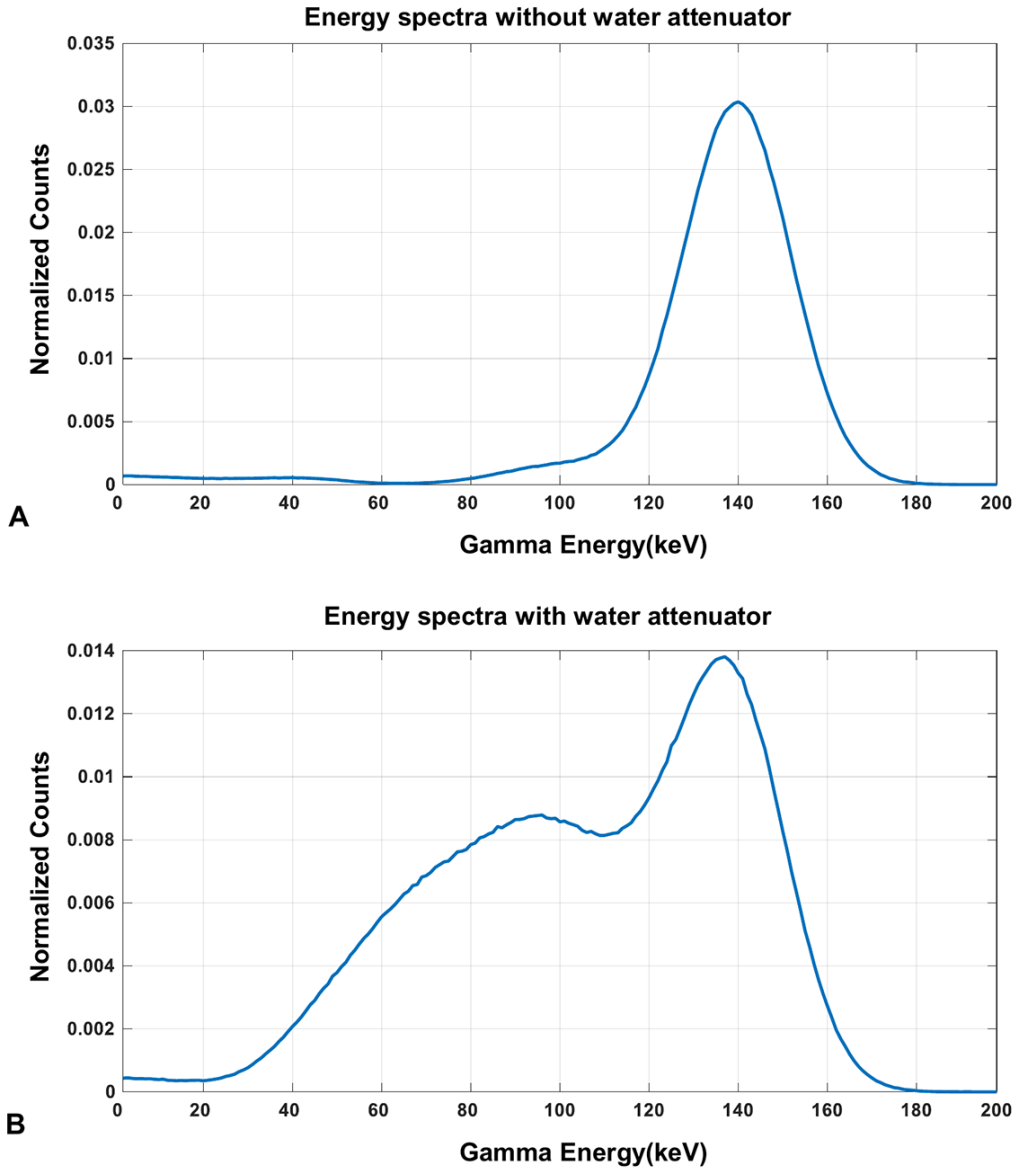
The energy spectra of ^99m^Tc source measured in the simulation **(A)** without the water-filled cylindrical attenuator and **(B)** with the water-filled cylindrical attenuator in the FOV.

The energy spectra of ^99m^Tc from the simulation is shown in Figure 9. From the results, the characteristic photo-peak is identified at the correct position clearly without the water-filled cylindrical attenuator in the FOV, while the photo-peak shifts to the left due to the scatters with the attenuator.

**Figure 10:**
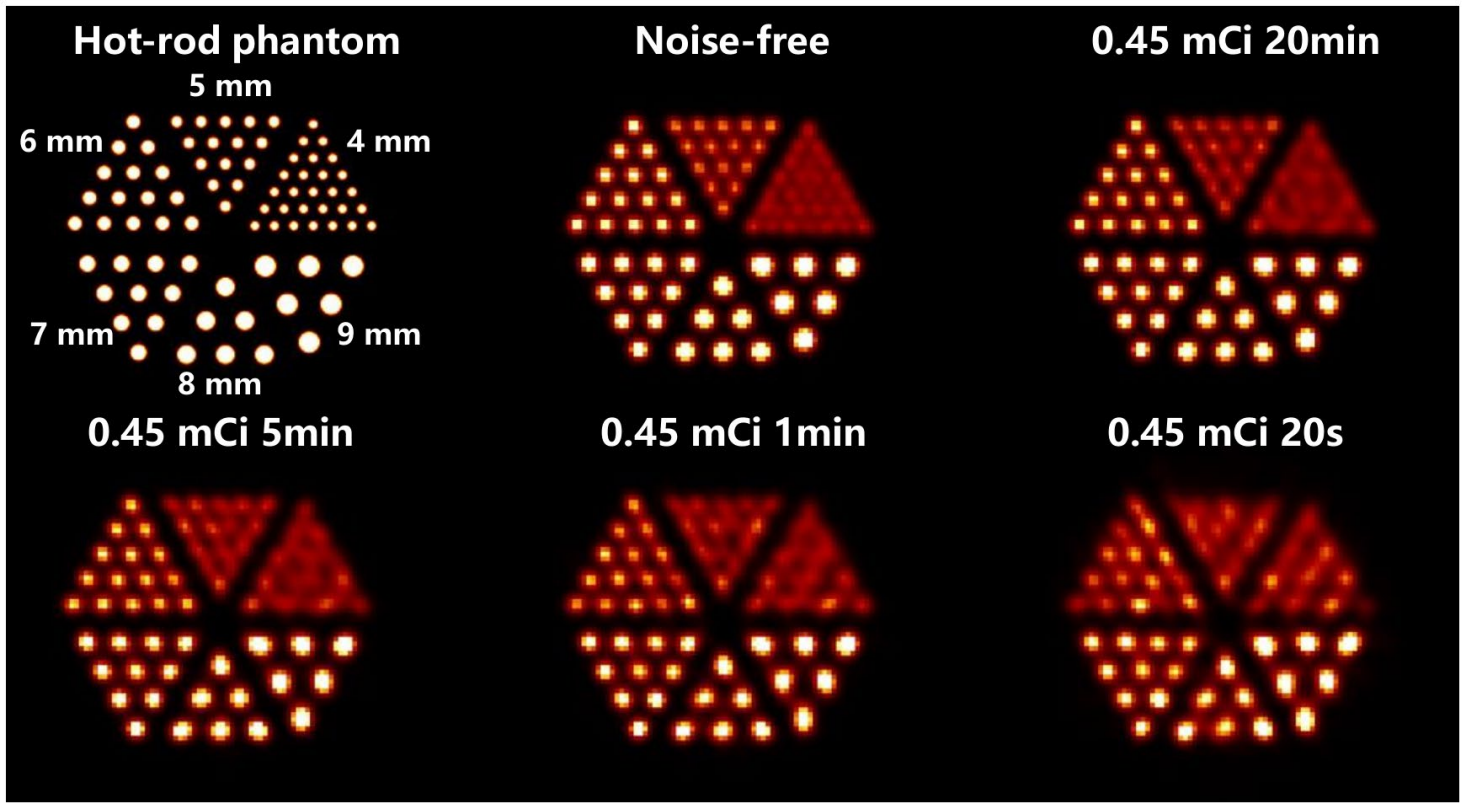
Reconstructed images of the hot-rod phantom using OS-EM reconstruction algorithm in different imaging cases.

**Figure 11:**
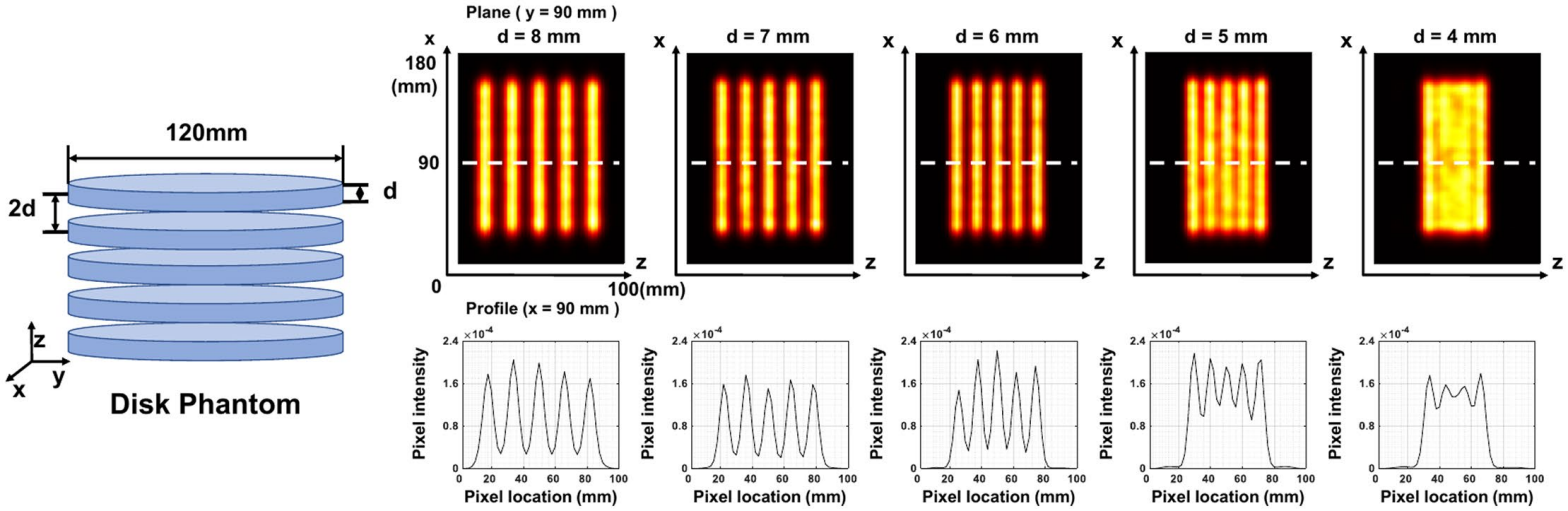
Reconstructed images of five disk phantoms with different disk thicknesses. The center of the image is at (90,90,50) mm. The plane image is at y = 90 mm, and the corresponding z-profile is at x = 90 mm.

Figure 10 demonstrates the in-plane resolution performance. With an acquisition time from 20 min down to 1 min, the hot-rod image has similar quality compared with the noise-free case, with the hot rod sections from 6 mm to 9 mm all separable. With 20 s acquisition, the reconstructed image shows visible distortion, however, the 6-mm hot rods are still visualized. In Figure 11, The disk phantom with 5-mm disk thickness and 10-mm disk separation is clearly separable.

**Figure 12:**
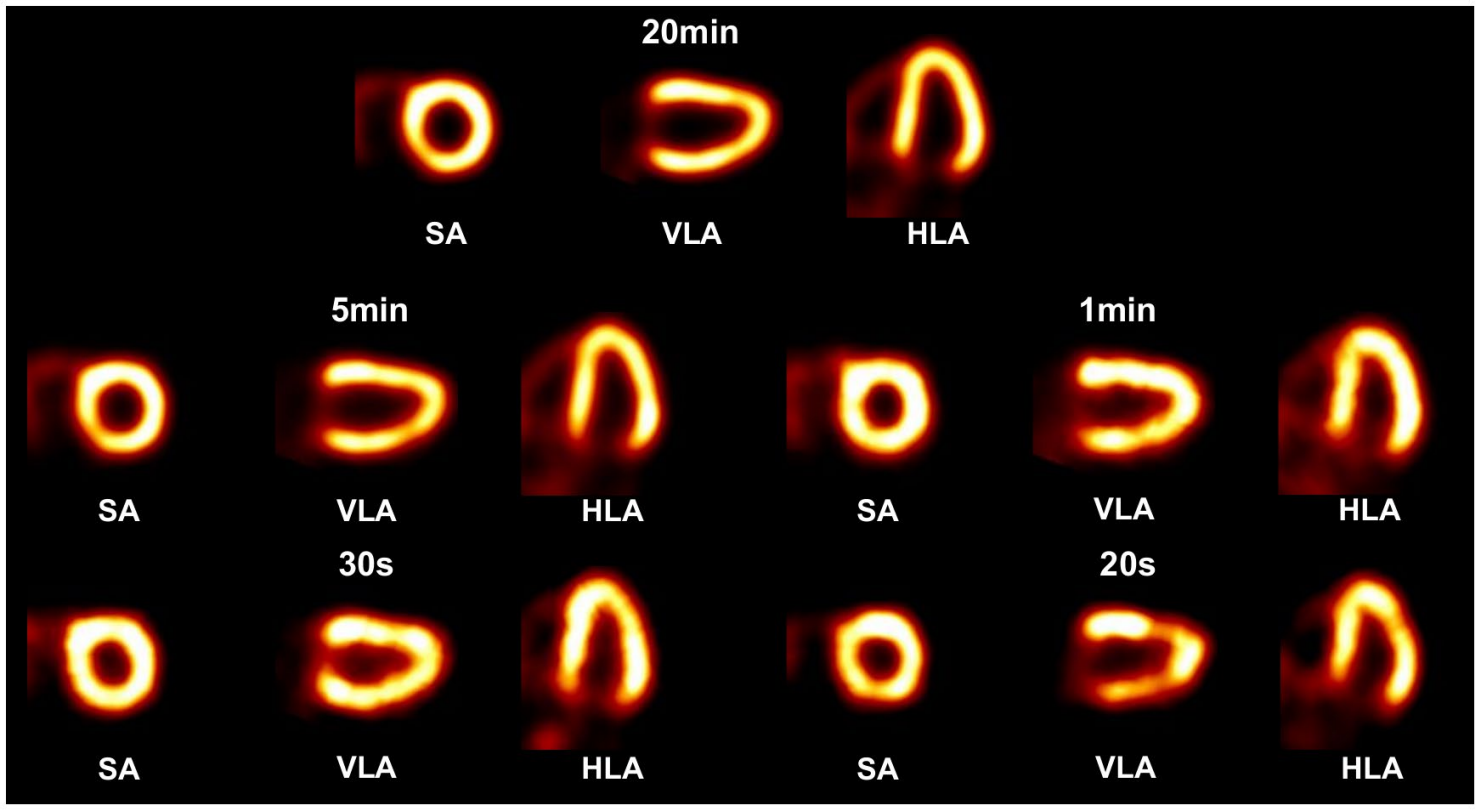
Reconstructed images of a 3-D cardiac phantom in short-axis (SA), vertical long-axis (VLA), and horizontal long-axis (HLA) using OS-EM algorithm in different scan time cases.

In the cardiac phantom study (Figure 12), the images with 20 min and 5 min acquisition time show clear contour and evenly distributed activity in the left ventricular myocardial region. When the acquisition time is shortened to 1 min and 30 s, slight image quality degradation is observed, but the radioactivity distribution in the left ventricular myocardial region is still satisfactorily reconstructed. The image with 20 s acquisition shows noticeable activity discontinuity in the myocardium.

### 3.2. Experimental prototype system

**Figure 13:**
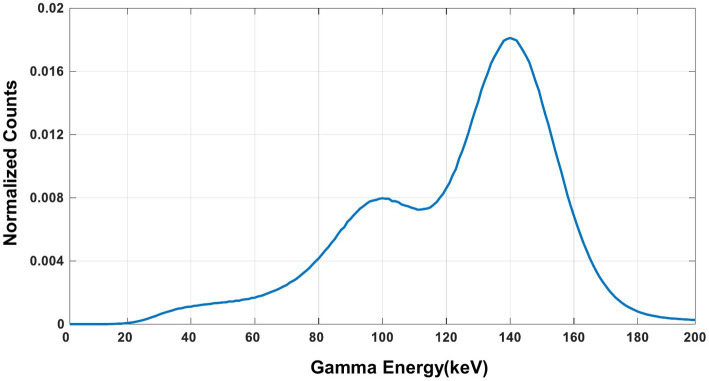
Energy spectra of ^99m^Tc measured in the experiment.

The experimentally measured energy spectra of ^99m^Tc is shown in Figure 13. The characteristic photo-peak is identified at the correct energy position clearly.

**Figure 14:**
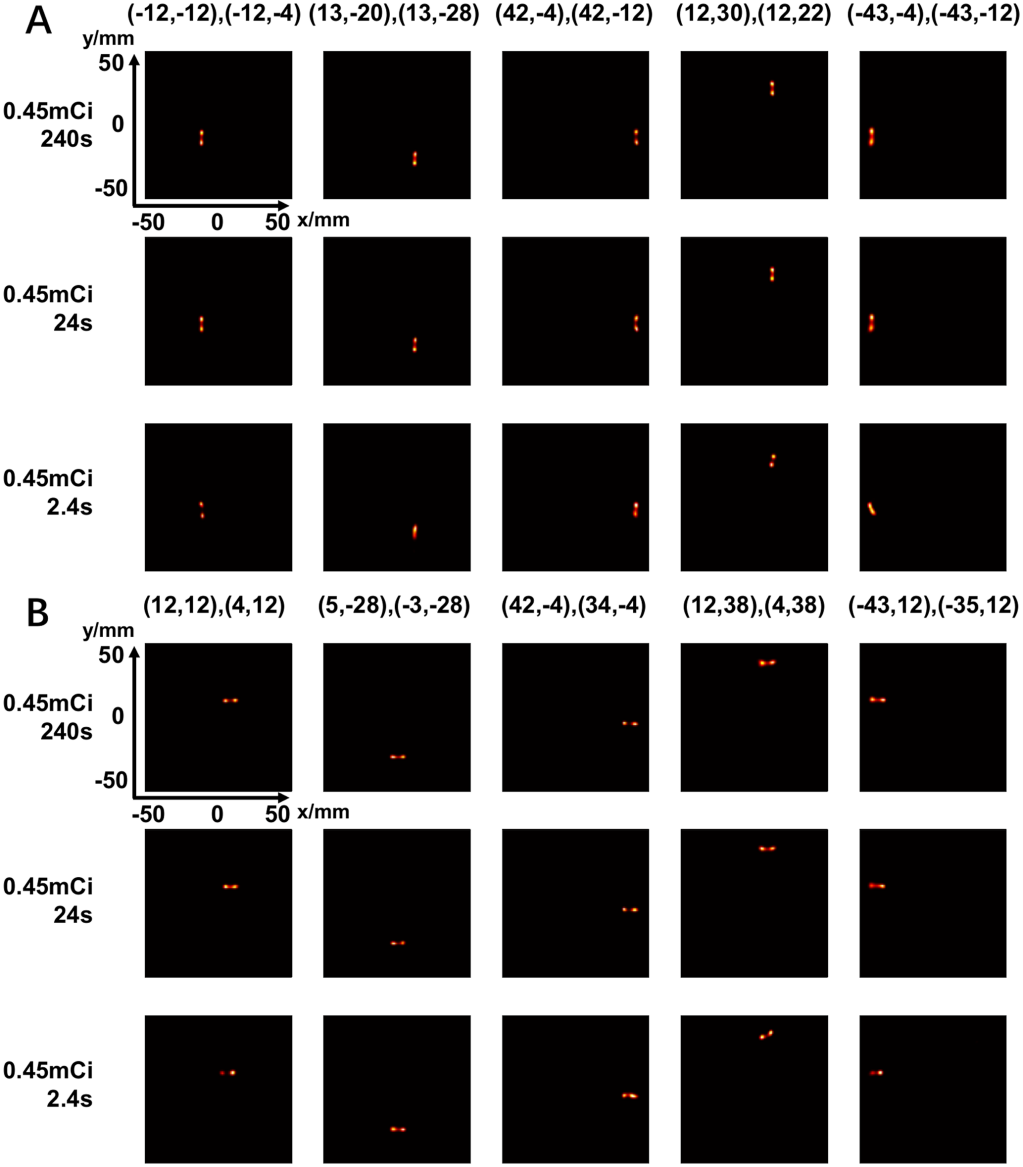
Reconstructed images of two-point-source placed at different positions in vertical directions **(A)** and horizontal directions **(B)** under different scan time (240 s, 24 s, 2.4 s) (unit: mm).

Figure 14 shows the reconstructed images of two point sources aligned in vertical (Figure 14A) and horizontal (Figure 14B) directions and placed at different positions in the FOV. The center-to-center distance of the point sources is 8 mm, and the diameter of each source is 0.68 mm. With an acquisition time of 240 s and 24 s, the point sources are separable at all the positions tested. With 2.4 s acquisition time, the two-point sources are undistinguishable at several positions.

**Figure 15:**
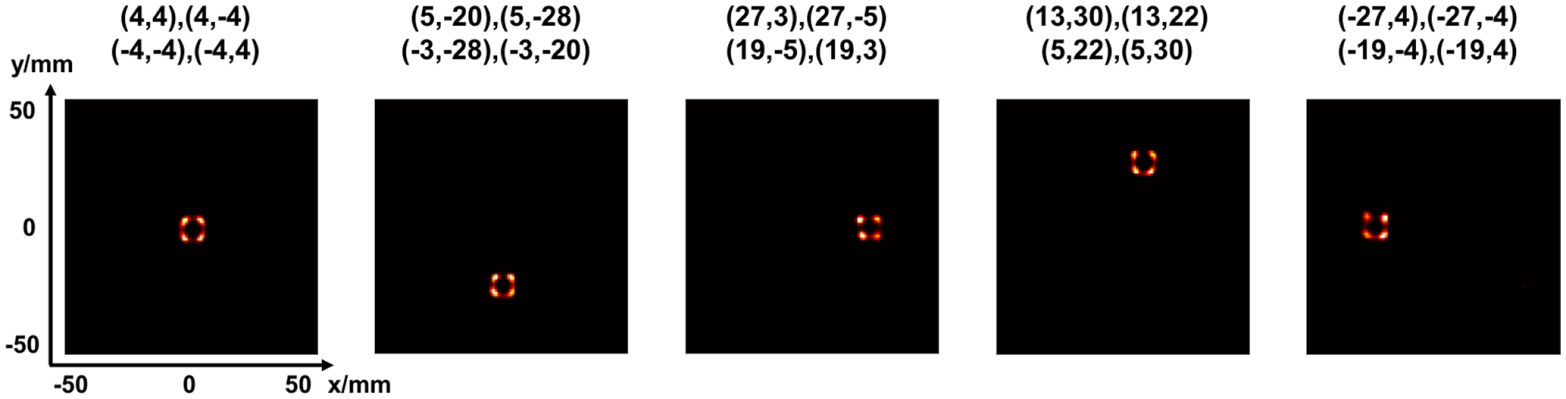
Reconstructed images of four-point-source at five positions in the FOV (unit: mm).

Figure 15 shows the reconstructed images of a 2-by-2 point source array placed at different positions in the FOV. The center-to-center distance is 8 mm. In all the cases, the point sources are clearly separable.

## 4. Discussion

In this study, we propose a novel cardiac SPECT imaging system design with only scintillators and without heavy metal materials. The key innovation is the mosaic-patterned scintillator assembly that allows one scintillator to be naturally collimated by other scintillators in front of it, enabling the determination of a photon’s pathway without sacrificing other photons as a mechanically collimated SPECT usually does. Therefore, it is possible to significantly improve the imaging speed for cardiac SPECT imaging.

Current work and two previous studies (33, 41) from our lab are under the same “self-collimation” concept but with different objectives and system setups. The main contribution of this work — different from the work in (33) is that we for the first time demonstrate the feasibility of tomographic imaging using a collimator-less mosaic-patterned scintillator block in this work. In contrast, the study in (33) reports imaging performance in a spherical plane in the far field, which is fundamentally different from tomographic imaging where line integrals through image voxels are involved. This work implements the concept of using scintillators as collimators in a fundamentally different way compared with the approach described in (41). Here, a collimator-less imaging system is used, and the 3D photon position information is acquired using dual-end-readout technology for the mosaic-patterned scintillator. In contrast, the detectors in (41) are assembled as multiple separate layers, with photon position information read out using a single SiPM array on the back side and a metal layer placed between the object and the first detector layer. As a result, there are significant differences in imaging system design and performance between the two approaches.

We have investigated the feasibility of simulation and proof-of-concept experiments. In simulations, we designed a half-ring cardiac SPECT with 7 mosaic-patterned detector modules and demonstrated that the system offers satisfactory image quality at routine tracer injection dose levels clinically. The image resolution is comparable to state-of-the-art dedicated cardiac SPECT systems. The reported sensitivity in the designed system (16.31% ± 8.85% in a 180 mm (Φ) × 100 mm (L)) is extremely high. However, since the collimation geometry and detection efficiency vary from one scintillator to another in a complicated way, one cannot simply compare the absolute sensitivity to a conventional SPECT with parallel-hole or pinhole collimation. Further research is required to explicitly analyze the image signal-to-noise property of the collimator-less SPECT system. However, the cardiac phantom study in Figure 12 shows reasonably good MPI images acquired in 30 s, which suggests a significant imaging speed improvement with the proposed system. In the experiments, we have successfully acquired artifact-free images of multiple point sources across the FOV. To the best of our knowledge, this may be the first cardiac SPECT image that is generated without a heavy-metal collimator. The experimentally achieved in-plane image resolution performance is comparable with existing dedicated cardiac SPECT scanners.

Our imaging system relies on photons interacting with the scintillator material, potentially producing scattered events across multiple scintillators via Compton scattering. However, our Monte Carlo simulations indicate that the detected Compton-scattering events in our proposed scintillator block are approximately 9.4% in an energy window between 112 keV and 168 keV. Of these events, around 3% result in inter-crystal scatter events, making the impact of scattering events insignificant in our system.

This study has several limitations. Firstly, the cardiac phantom study shows that scattering events do not significantly degrade image quality in a uniform water phantom of fixed size. However, we did not consider the impact of non-uniform body attenuation and scattering in our simulations. Scattering can have a more pronounced effect on patients with a large BMI. Secondly, the residual tracer activity out of the FOV (such as liver) is not included as well, which may cause further image quality degradation. Thirdly, our currently available detector only implements a mosaic-patterned design in the trans-axial direction, which differs from the proposed imaging system design. Furthermore, the experiments we conducted were in a planar field of view. Further work is ongoing to assemble a 3-D imaging system that matches the simulation design and investigate the impact of non-uniform body attenuation and scattering in different patient sizes.

## 5. Conclusion

We propose a novel collimator-less cardiac SPECT system by using a mosaic-patterned scintillator block design that allows detector collimation through other detectors in the front. We propose a half-ring cardiac SPECT design with 7 mosaic-patterned detector modules. The simulation study demonstrates that 6-mm hot rod separation and super high detection efficiency (16.31 ± 8.85%) are achievable. The prototype experiment demonstrates the feasibility of multi-point-source imaging with an 8-mm point-source separation capability. The proposed cardiac SPECT allows myocardial SPECT scan in less than a minute with a highly flexible and scalable system structure. We conclude that it is possible to achieve high-performance SPECT imaging without a heavy-metal collimator, and our work opens the way to a very fast SPECT MPI scan with reasonable resolution.

## Data availability statement

The raw data supporting the conclusions of this article will be made available by the authors, without undue reservation.

## Author contributions

RW: methodology, validation, formal analysis, investigation, and writing – original draft. DZ: methodology and formal analysis. YH: methodology and investigation. ZL: project administration. TM: conceptualization, supervision, writing – review and editing, and funding acquisition. All authors contributed to the article and approved the submitted version.
